# Multilayer Approach for Product Portfolio Optimization: Waste to Added-Value Products

**DOI:** 10.1021/acssuschemeng.1c01284

**Published:** 2021-04-28

**Authors:** Lidia S. Guerras, Debalina Sengupta, Mariano Martín, Mahmoud M. El-Halwagi

**Affiliations:** † Department of Chemical Engineering, University of Salamanca, Plz. Caídos 1-5, 37008 Salamanca, Spain; ‡ Gas and Fuels Research Center, Texas A&M Engineering Experiment Station, 7607 Eastmark Drive, College Station, Texas 77840, United States; § Artie McFerrin Department of Chemical Engineering, Texas A&M University, 3122 TAMU, 100 Spence Street, College Station, Texas 77843, United States

**Keywords:** food−water−energy nexus, multiproduct facility, superstructure optimization, olive waste valorization

## Abstract

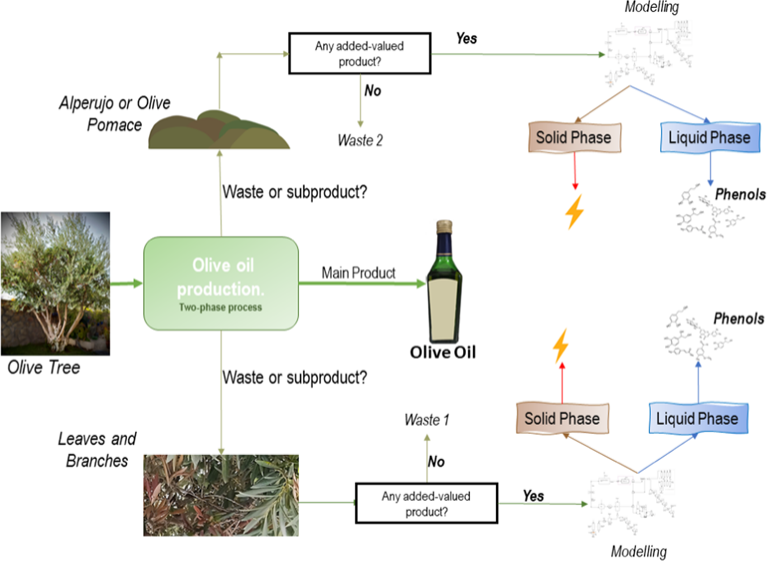

A multistage multilayer systematic procedure has been developed for the selection of the optimal product portfolio from waste biomass as feedstock for systems involving water–energy–food nexus. It consists of a hybrid heuristic, metric-based, and optimization methodology that evaluates the economic and environmental performance of added-value products from a particular raw material. The first stage preselects the promising products. Next, a superstructure optimization problem is formulated to valorize or transform waste into the optimal set of products. The methodology has been applied within the waste to power and chemicals initiative to evaluate the best use of the biomass residue from the olive oil industry toward food, chemicals, and energy. The heuristic stage is based on the literature review to analyze the feasible products and techniques. Next, simple metrics have been developed and used to preselect products that are promising. Finally, a superstructure optimization approach is used to design the facility that processes leaves, wood chips, and olives into final products. The best technique to recover phenols from “alperujo”, a wet solid waste/byproduct of the process, consists of the use of membranes, while the adsorption technique is used for the recovery of phenols from olive leaves and branches. The investment required to process waste adds up to €110.2 million for a 100 kt/yr for the olive production facility, while the profit depends on the level of integration. If the facility is attached to an olive oil production, the generated profit ranges between 14.5 MM €/yr (when the waste is purchased at prices of €249 per ton of alperujo and €6 per ton of olive leaves and branches) and 34.3 MM €/yr when the waste material is obtained for free.

## Abstract 2

Methodology and case study for the selection of added-value products out of waste and process design.

## Introduction

The term water–energy–food (WEF) nexus was coined back in 2011 in the World Economic Forum to strengthen the links among different resources and to provide basic and universal rights to food, water, and energy security.^1^ Since then, the WEF nexus has gained increasing attention globally from different perspectives (e.g., engineering, social sciences, policy) due to their critical importance toward sustainable development. A key challenge in addressing the WEF nexus is the need to consider the strong interactions among the various components. For example, zones of drought have food and energy security problems because of the high consumption of water supply involved in their production.^2^ The energy, food, and water demand has increased in the world because of the increase in the population. Therefore, there is a growing need for improving the efficient use of resources within the WEF nexus. One important point to promote and strengthen the WEF nexus is to employ the circular economy approach. One example is the case of the agricultural sector, in particular, the food industry, that generates a large amount of waste that can generate greenhouse gases or pollute water due to the decomposition of the residues. If such wastes were used as raw materials (RM) to produce added-value products or to generate energy, they become a valuable resource. Thus, the circular economy concept is a strategy that improves the economics and the environmental impact^3^ as it can be seen in previous cases in the orange^4^ and coffee^5^ industries and the production of biofuels.^6^

In the case of processing olives to produce olive oil, several wastes are produced including branches, leaves, and a wet solid lignocellulosic material called “alperujo”. Recently, 19–20 million tons of olives per year have been harvested around the world. Spain is the largest producer with estimated shares ranging between 25 and 40% of the world’s production and 41 and 72% of the total amount of olives produced in Europe. Greece and Italy follow Spain as major world producers representing 8–16 and 11–18%, respectively.^7^ In Europe, 50–70% of the olive production is used to obtaining olive oil, while the rest is used to develop different types of table olives. In Spain, 90–94% of the amount of olive harvesting is employed to produce olive oil.^8^

Since ancient times, olive oil and diverse types of leaves and branches have been used to produce various types of foods, medicines, perfumes, and fuels. Recently, attention has been devoted to the valuable components contained in olive milling wastes including phenols, organic matter (nitrogen, phosphorus, potassium), and sugars, among other species. Several works have evaluated the recovery of phenols and compared alternative processing techniques for the different types of residues such as alperujo, leaves, and branches. These processes can be classified mainly into two groups, namely, conventional and modern techniques. The advantages of modern techniques include a lower time of extraction, easier scale-up to industrial scale, and less use of solvents among others.^9^ Most of the previous studies have evaluated the recovery of value-added products from an experimental point of view to determine the yield and operating conditions. In the case of membrane module, different combinations of membranes are used to recover distinct classes of phenols. Examples of such systems include microfiltration, nanofiltration, and osmotic distillation membrane to recover phenols in general^10^ or the combination of microfiltration, ultrafiltration, and nanofiltration membranes for the recovery of oleuropein from olive leaves and branches.^11^ Adsorption technique was employed using different types of resins and operation conditions. For instance, Yoon et al. studied the adsorption of flavonoids employing the XAD-7 resin,^12^ while Aehle et al. used XAD-7HP and XAD-16HP resins.^13^ Bayçın et al. used a silk fibroin to recover the antioxidants, analyzing different parameters like temperatures, pH, and solid/liquid ratios.^14^ The work of Li et al.^15^ evaluated eight types of resins to purify phenols and flavonoids from olive leaves. There are other studies that compare conventional and modern techniques in the recovery of phenols such as Lama-Muñoz et al. that compared the Soxhlet method to the pressurized liquid extractor (PLE)^16^ or optimized the phenolic compound extraction through the pressurized liquid extractor by changing the operating conditions.^17^ Works by Serrano et al. presented several economic evaluation studies about different pretreatments and recuperation of phenols after biomethanization of the wastes.^18−20^ Lama-Muñoz optimized the operating conditions of pressurized liquid extraction, developing correlations to determine extraction yields, total phenol content, total flavonoid content, as well as oleuropein content as a function of moisture content, temperature, and ethanol concentration.^21^ Therefore, systematic methods for product selection and process design are still missing in the literature.

A systematic methodology is proposed for the selection of a set of promising products out of waste biomass to identify and select a subset and the techniques to recover them. It is applied to a particular case of study. This work aims at the development of a process for the recovery of a limited set of added-value compounds from diverse olive oil wastes, including leaves and branches. The rest of the paper is organized as follows. The next section presents the proposed methodology, followed by the general description of the formulated superstructure, associated modeling of the units, and the solution procedure for the problem are shown. Subsequently, the results are analyzed and finally the conclusions are drawn.

## Methodology

The methodology consists of two stages. The first one deals with the prescreening of potential products. We define different indicators to be able to rank the high added-value products available within the biomass. Once a subset of chemicals is selected, a superstructure optimization approach is formulated to select the techniques and the optimal portfolio of products.

### Product Prescreening

Four different metrics are developed to quickly identify potential promising products contained or extractable from the biomass. The indicators are ranked so that first the economic potential is evaluated. Next, the process conditions are evaluated. The larger the energy required to obtain a particular product, the higher is the associated operating cost. The third layer of decision-making is given by the operating conditions of the process. The more extreme conditions require processing units to withstand them and increase the capital investment of the process. Finally, at the same decision level are the safety and health issues of the species involved in the processing of the waste for sustainable decisions.

### Economic Potential Indicator

From a benefit point of view, this indicator based on the metric for inspecting sales and reactants “MISR”^22^ aims to identify the type of compound that is more promising from the economic perspective. This indicator is computed as the ratio between the benefit from a particular product (P) and the cost of the raw material (RM) (see eq 1)
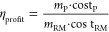
1This indicator ranks chemicals as a function of their economic potential, where a value greater than 1 indicates economic feasibility of the product, while the greater the indicator is from 1, the more profitable the pathway is expected to be.

### Energy and Environment

The biomass may be converted into products. The smaller molecular size of the final product or required intermediates such as syngas require larger energy and number of units for processing. The ratio of the molecular size of the product versus that of the raw material is representative of the energy involved as well as the equipment required. Therefore, the energy indicator, η, is computed as the ratio between the molecular weights (eq 2)
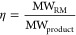
2The particular case of biomass presents challenges to compute this indicator. Hemicellulose, cellulose, and lignin are mostly polymers. Therefore, to estimate the molecular weight of the initial raw material, the degree of polymerization (DP), by number, is used
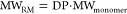
3Hemicellulose shows degree of polymerization (DP) values of 100–200,^23^ cellulose shows 700,^24^ and lignin shows a value of 60.^25^

When dealing with lipids, the composition of the lipid as a mixture of them allows computing the average molecular weight from the distribution of fatty acids. The unit of measure is Da (= 1 g/mol). In some cases, the final product is already available as such within the raw material and only extraction is required. In that case, this indicator becomes 1.

The operating conditions to process the biomass into products can also be classified by high-, medium-, and low-temperature processes such as gasification, pyrolysis, and biochemical pathways. Each of them has its own typical equipment and costs. Therefore, the maximum operating temperature and pressure across the process are also metrics to indirectly evaluate the environmental impact and safety issues of the transformation process. Operating indicators *m_P_* and *m_T_* are defined as given in eq 4
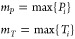
4The maximum temperature can also be related to the emissions since various energy sources are required to provide high temperatures. Some examples are shown in Table 1.

**Table 1:** Temperatures of Typical Biomass Processing Routes and the Sources of Energy

temperature (°C)	process type	source of energy	emissions
1000	gasification	combustion	kg CO_2_/kg biomass
500	pyrolysis	combustion/CSP	
250	high-temperature biochem	HP steam	
180	medium-temperature biochemical	MP steam	
120	medium- to low-temperature biochemical	LP steam	
<100	low temperature	LP steam/energy integration	

To further distinguish among processes operating at the same temperatures and pressures, for instance, among pretreatments in lower-temperature processes, the need for adding an acid, a base, or a solvent is an additional feature to characterize the process. Solvents can be flammable, explosive, and/or toxic. Martínez-Gomez et al.^26^ presented a study where the safety of a heat transfer fluid was analyzed. For quick and preliminary assessment of techniques, the processes that involved chemicals with the highest LC50 will be considered last or discarded. A safety analysis can be part of the detailed process design at the superstructure optimization stage if needed.

### Superstructure Design

Based on the subset of promising products, a superstructure of alternatives is formulated to design the production process capable of recovering them.

### Heuristic-Based Selection of Techniques

We consider different raw materials arising from the production of olive oil. On the one hand, during the harvest, leaves and branches can be collected. On the other hand, once the oil is extracted from the olive, the alperujo or olive pomace is the second waste that becomes a raw material of the process. The superstructure will consider both processing lines.

Several methods to obtain olive oil are employed traditionally including pressing and centrifugation. Nowadays, centrifugation methods are employed more at the industrial scale because they can work in continuous processes.^27^ Lately, two-phase and three-phase centrifugation are the more widely used. The two-phase centrifugation process uses less water and generates the least residues compared to the three-phase centrifugation process. Because of this, in Spain, 90% of the production of olive oil is carried out by the two-phase method.^28^ This will be the technique of choice in this work.

**Figure 1:**
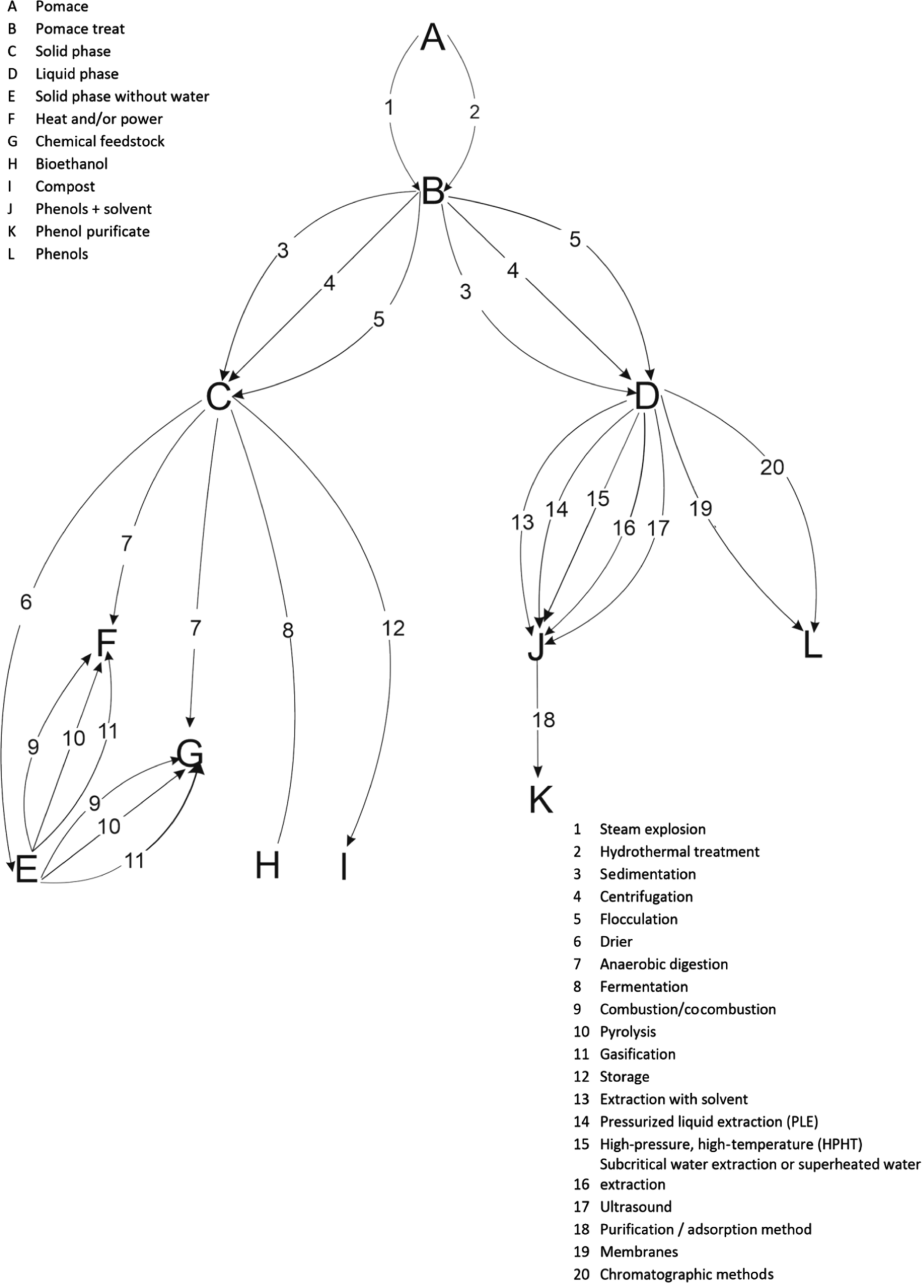
Techniques to recover high added-value compounds from olive pomace.

Alperujo is composed of a mix of biomass and water as well as a solid fraction consisting of bones, mesocarp, and skin. Alperujo has a high organic content, and it presents phytotoxic components. This fact generates environmental problems and makes it difficult to use.^29^ One important feature of alperujo is that it is not possible to separate both phases by centrifugation, decantation, or another process from the beginning. To perform this separation, it is necessary to apply a preprocessing or pretreatment stage.^30^ The main technique is thermal pretreatment and usually consists of steam explosion and hydrothermal treatment.^31^ Thermal pretreatment is necessary to solubilize the phenolic compounds in the liquid phase by means of their breakdown from complex molecules.^32^ Next, it is possible to carry out the phase separation. In the case of industrial scale, the hydrothermal treatment is better than steam explosion because of the lower operating temperatures and pressures.^30^ Sedimentation, centrifugation, or flocculation can be employed for the separation between the liquid phase and the solid phase. At industrial scale, the best process to achieve this separation is centrifugation due to fast splitting, avoiding the use of chemicals as in the case of flocculation.^33^ At this point, the liquid phase follows one path and the solid phase another. It should be noted that the liquid phase contains most of phenols within the alperujo. Due to this fact, the solid phase is not employed as a resource for phenols but it can be used to generate other chemicals such as biomethane^18^ or bioethane,^34^ power or heat,^34^ and compost.^35^ In some cases, the liquid phase can be used for irrigation. However, in this work, the target is to obtain added-value compounds, the phenolic ones, due to their wide array of biological activities.^36^ Different techniques exist for the recuperation of these phenols. The first technique, also called a conventional technique (Soxhlet and maceration), is based on the extraction of phenols using different solvents. Depending on the nature of the compound to be obtained, the type of solvent is employed.^33^ The main problem with these techniques is the time of extraction to obtain high yields of phenols. Advanced extraction techniques allow decreasing the time of extraction and energy consumption. These types of techniques can be classified into pressurized liquid extraction, high-pressure or high-temperature, subcritical water extraction or superheated water extraction, supercritical fluid extraction, molecular distillation or short-path distillation, ultrasound-assisted extraction, microwave-assisted extraction, chromatographic methods, or membranes.^36^ In this case, use of membrane module is a good technique for recovering the phenols due to their low cost and low consumption of energy.^37^ Furthermore, this technique not only recovers the phenols but also produces them with the required degree of purity.^38^ From the industrial point of view, there is another interesting technique to recover phenols, the adsorption technique or the chromatographic technique.^39^ Based on the species-pathway representation of Pham and El-Halwagi,^40^Figure 1 shows the possibilities for the recovery of phenols from alperujo based on the above discussion.

**Figure 2:**
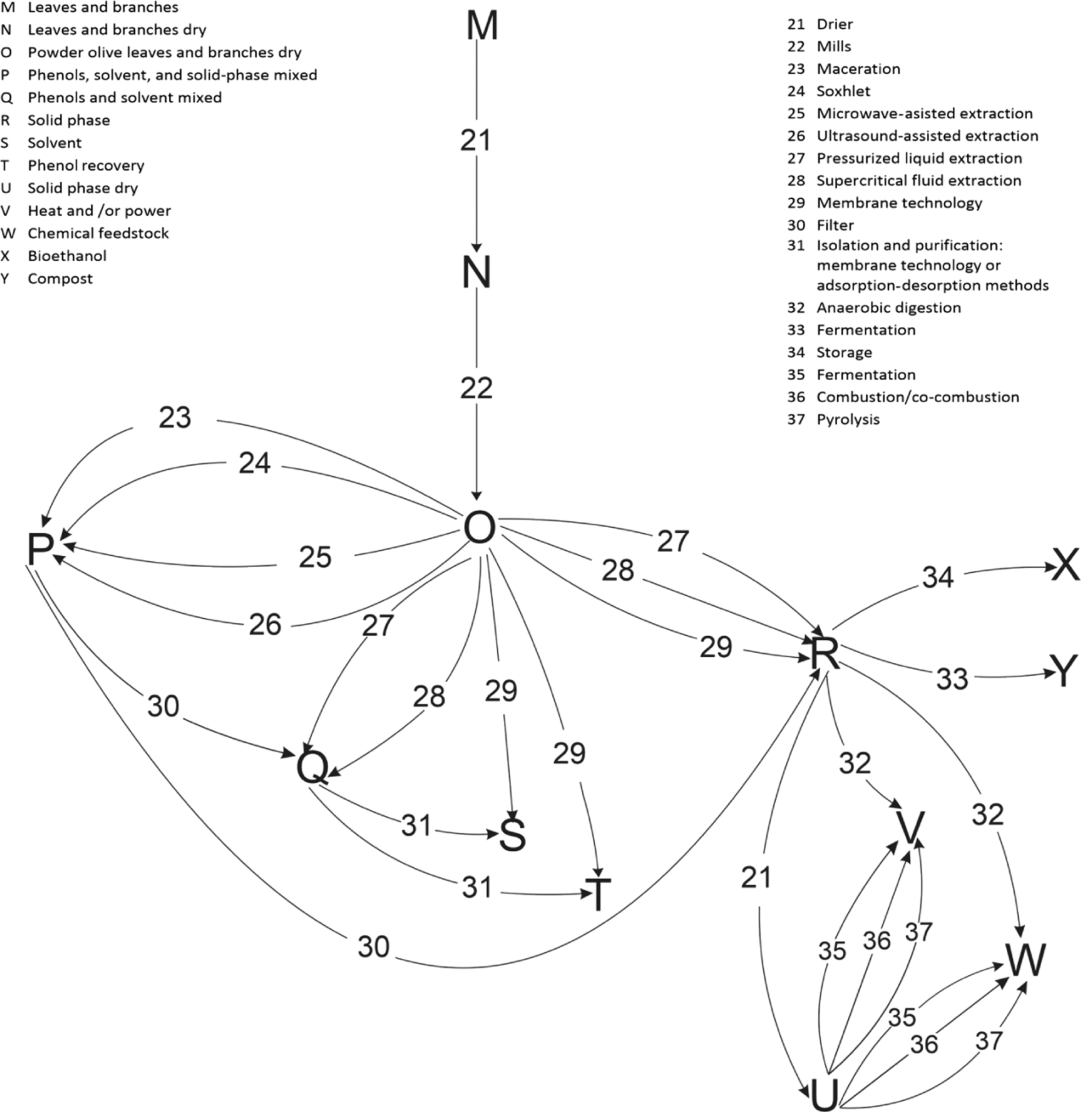
Techniques to recover high added-value compounds from olive leaves and branches.

Over the last years, different techniques have been developed to extract phenol compounds from olive leaves. Maceration and Soxhlet extraction are very popular ones but the main problem is that they are difficult to scale up to the industrial scale. They are used mainly at the laboratory scale.^9^ Other techniques such as pressurized liquid extraction, dynamic ultrasound-assisted extraction, supercritical fluid extraction, or microwave-assisted extraction have been evaluated.^41^ Among the modern techniques, the pressurized liquid extraction process is the more promising technique for recovering polyphenols from olive leaves^42^ and it was also successful for phenol recovery from various plant matrices and can be used at the industrial scale.^43^ After applying this technique, product purification has to be carried out. Two alternatives can be used, either membrane modules or adsorption–desorption beds. Figure 2 shows processes that can be applied to recover added-value products from leaves and branches.

### Superstructure Definition

**Figure 3:**
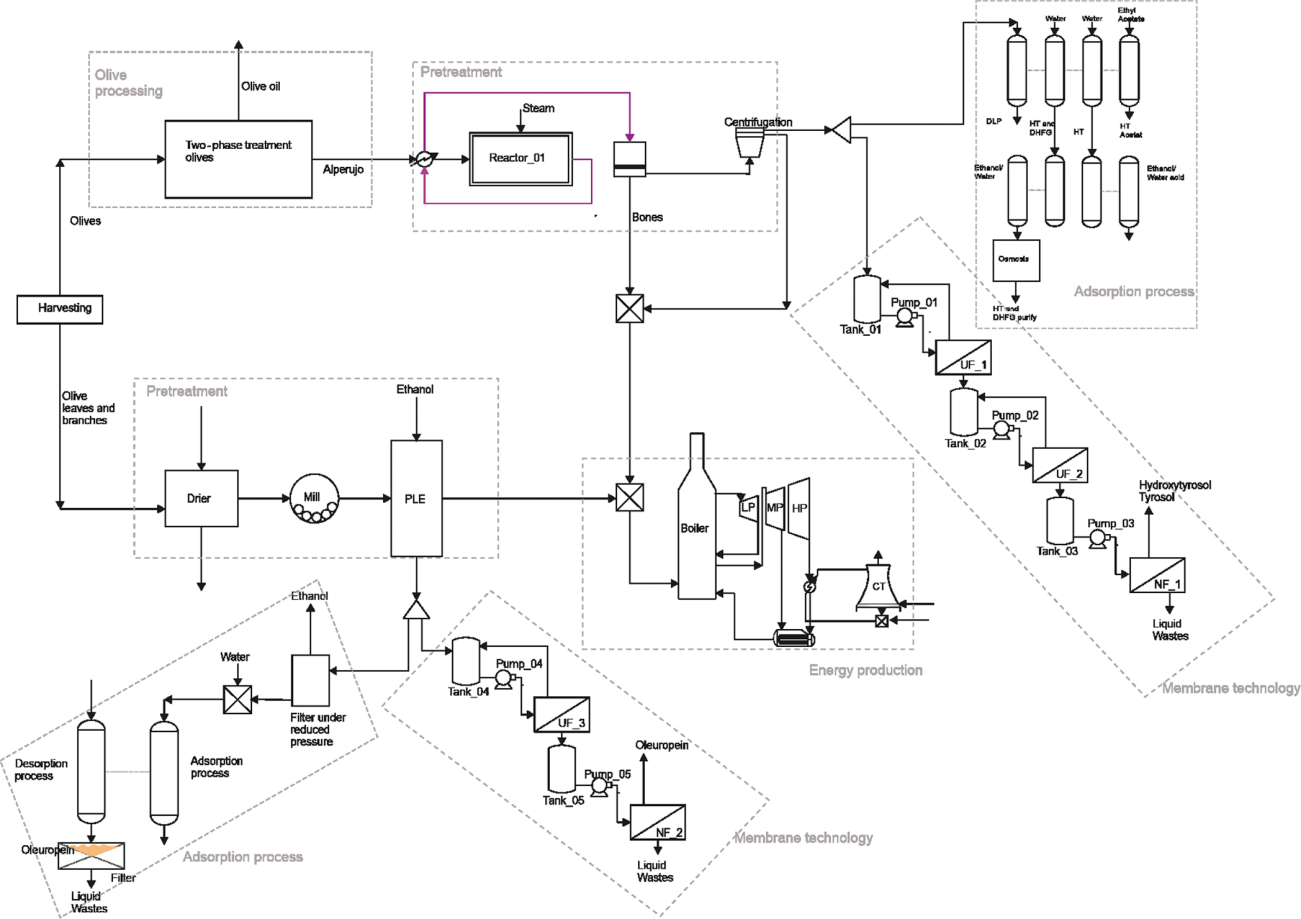
Superstructure of alternative techniques for the recovery of phenols from olive pomace, olive leaves, and branches and recuperation of energy.

As referenced above, after harvesting, cleaning, and production of olive oil, mainly two types of wastes are generated, alperujo (the liquid waste stream from oil extraction) and leaves and branches. These wastes are treated separately for the recovery of phenols. Figure 3 shows the superstructure including the most promising techniques. The harvesting of olive and generation of olive oil are carried out for around 4 months. In this way, to develop the model of the facility, the olive leaves and branches are processed for 8 months, while the alperujo is processed for 4 months. During these 4 months, the olive leaves are dried, crushed, and stored. These treatments are necessary to avoid the degradation of olive leaves.

In the case of alperujo, the first step is the hydrothermal treatment. After the pretreatment, the liquid and the solid phases are separated. The solid phase is used for the generation of energy using a boiler and a steam turbine and the liquid phase is used to recover phenols. The recovery of phenols can be carried out through two techniques, either membrane modules or adsorption–desorption techniques.

In the case of leaves and branches, the first step consists of drying, milling, and storage. For the recuperation of phenols, the pressurized liquid extractor (PLE) is selected, using ethanol as a green solvent. In this process, liquid and solid phases are generated. Similar to the processing of alperujo, the phase solid is employed to generate energy, and the liquid phase is employed as a raw material for the recovery of phenols. To purify the phenols soluble in the solvent, either membranes or adsorption–desorption techniques are used.

The solid phase is burnt to generate energy through a regenerative Rankine cycle with reheating.^44^

## Superstructure Model Formulation

The superstructure described in the previous section, and presented in Figure 3, is modeled in this section. The modeling of the superstructure is developed unit by unit, considering mass and energy balances, thermodynamic properties, and experimental data in the appropriate unit. Surrogate models are developed from experimental data. To simplify the explanation of the superstructure, the selection of the products based on the indicator should be mentioned; for more information about this analysis, we refer to the Portfolio of Products section in this work. Therefore, the phenols recovered from alperujo are hydroxytyrosol and tyrosol, while from olive leaves and branches, it is oleuropein. In addition, the explanation of the superstructure is divided into three parts for the detailed presentation of the models developed. All of the units are modeled based on mass and energy balances using experimental yields from the literature, as will be explained in the following sections.

### Recovery of Phenols from Alperujo

The composition of alperujo depends on the olive’s variety, the fruit ripeness, among other parameters; in such a way, it is widely variable.^31^Table 2 shows the composition of alperujo employed in this work.

**Table 2:** Main Compounds of Alperujo^a^

component	% wet weight
bones	5.062
orujillo	17.084
WSC	0.028
phenols	0.066
hydroxytyrosol	0.120
tyrosol	0.060
oil	4.081
water	73.500

a Adapted.^39,45^ WSC, water-soluble content.

### Pretreatment of Alperujo

The first stage is the pretreatment of the alperujo (see the top line in Figure 3). The process used for the pretreatment is based on the patent number WO 2012/020159 A1. The process developed in this patent consists of one heat exchanger, a reactor, the split of bones, and a centrifugation process (see Figure 3). The first step of the pretreatment is preheating the alperujo in a heat exchanger, using the hot stream exiting the reactor. Therefore, with this type of preheating, heat integration is achieved minimizing the consumption of steam. The preheated alperujo is sent to the reactor heating with steam. To provide the heating, 1.1 L of steam per kg of fed alperujo^18^ is used, assuming medium-pressure steam at 230 °C and 2.7 MPa. The reactor is equipped with a stirrer and a heating jacket. In the operation, the reactor operates around 170 °C and 0.85 MPa. The pretreatment increases the solubility of the organic compounds up to around 26.3% compared to the initial alperujo, and in the case of phenols, it reaches 60.4%.^18^

Once it has passed through the reactor, the separation of the solids and liquid phases is easier. Two types of techniques are used for such a separation due to the presence of two different particle sizes of the solids. A sifter is first employed to remove the bones broken in the process of crushing. Thus, two streams are generated, the bones with moisture and the rest of compounds. Generally, by the end of the process, the bones have an average of 13% moisture.^46^

To perform the separation process, a centrifugation system is employed. The solid-phase content is mainly orujillo, defined as the residue remaining after oil extraction from the oil, but around 25% of organic-soluble compounds or in the case of phenols 11.5% are lost in the solid phase. Besides, the content of water is around 8.7% of the product obtained.^47^ The liquid phase contains the majority of the organic-soluble compounds as well as phenols, so this stream continues the process of recovery of these phenols.

To compute the cost of this pretreatment, the data from Serrano et al. are employed and is under patent. In this study, 50.000 metric tons per year are treated, and the investment cost in the case of heat recovery reaches €330 000, while the operation and maintenance costs were estimated as 2% of the construction cost. Therefore, this investment cost is around 6.6 × 10^–3^ €/(kg yr).^18^ Therefore, the cost (eq 5) is calculated as follows
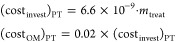
5

### Techniques for Phenol Recovery

In this work, two techniques to recover the phenols are considered, resin adsorption columns and membrane modules (see Figure 3); only one is to be used.

### Resin Adsorption Columns

One of the most popular alternatives to recover phenols is based on the patent WO 2013/007850 A1. This patent presents several adsorption and desorption steps through different columns of anionic and cationic resins. The liquid phase obtained in the previous separation is fed to the columns (see the top line in Figure 3). This process is carried out by floating or force of gravity. The phenols are retained in the bed of the columns, and to desorb them, different steps are followed. The first step consists of the discharge of the column with water, obtaining 3,4-dihydroxyphenylglycol and hydroxytyrosol. The second step is based on the discharge of the column with water at acidic pH, obtaining hydroxytyrosol with 30–70% of purity. In the end, the column is emptied, refilled with ethyl acetate, and closed. The temperature is increased with a heating jacket and then it is emptied; acetate hydroxytyrosol is obtained. According to Serrano et al., using this process, the yields of hydroxytyrosol, tyrosol, 3,4-dihydroxyphenylglycol, and vanillic acid are about 77.8, 93.0, 75.9, and 92.5%, respectively.^18^ It should be noted that in this case, hydroxytyrosol and tyrosol are recovered separately.

As in the previous case, this process is under patent, and to compute the cost, the data of Serrano et al. are employed.^18^ In this way, the investment cost is around 0.42 €/kg and the operation and maintenance cost is 0.5 €/(kg yr),^18^ as shown in eq 6
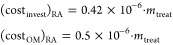
6

### Membrane Techniques

Another alternative employed to recover phenols consists of the use of membrane modules (see the multistage membrane system on the right of Figure 3). This process is based on the purification of the olive pomace. The small area requirement and low-energy consumption of the operation make it popular in water treatment and it is feasible for olive pomace treatment.^48^ In this work, this technique is based on three types of membranes, two ultrafiltration membranes and one nanofiltration membrane.

The first step of the process is to increase the pressure until a transmembrane pressure of 0.5 bar is achieved. The yield considered for the pump is around 80%. Once it has passed through the membrane, the permeate is sent to the next membrane using another pump. In the second step, using a second pump, the transmembrane pressure (Δ*P*) must reach 9 bar. The final step consists of recovering the phenols using the nanofiltration membrane. The permeate obtained from the second membrane is sent to the nanofiltration membrane using a third pump, achieving a transmembrane pressure of 12 bar. The parameters employed to develop the mass balance like yields, permeate flux, transmembrane pressure, and water permeability are given in Table 3.

**Table 3:** Properties of Membranes^49^

	*J*_p_ (L/(m^2^·h))	*K*_water_ (l/m^2^·bar)	η_phenols_ (%)	η_SCO_ (%)
UF 1	12	218.07	21	33.7
UF 2	150	22.47	17.6	72.1
NF	13.75	5.95	100	96.4

The temperature of operation employed in the process is the ambient temperature. It should be kept in mind that the membrane module does not recover phenols separately but a mix of phenols where the principal phenols are hydroxytyrosol and tyrosol.

In the case of membrane technique, three types of membranes are employed, two ultrafiltration membranes and one nanofiltration membrane. In the case of ultrafiltration membranes, the investment cost accounts for 5.37 €/m^3^ per year, while the operating costs are 0.54 €/m^3^ per year. The process to compute the nanofiltration membrane is the same as in the previous case; therefore, investment costs are 3.79 €/m^3^ and operating costs are 0.49 €/(m^3^ yr)^50^
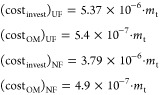
7

### Recovery of Phenols from Leaves and Branches

During harvesting of olives, around 5% by weight of olive leaves, with respect to olives, are recollected.^31^ At this point, different treatments are described for the recovery of phenols from the olive leaves and branches. As mentioned above, the main phenol content in the olive leaves is oleuropein.

The typical composition of the olive leaves that is employed in the model is shown in Table 4. The added-value products are extracted from the carbohydrate and crude fiber. The second line of processes in Figure 3 shows the processing of leaves and branches.

**Table 4:** Composition of Olive Leaves^51^

component	amount (g/100 g olive leaves)
protein	5.45
oil	6.54
carbohydrate	27.57
crude fiber	7
ash	3.61
water	49.83

### Drying of Olive Leaves

After cleaning the olives and removing the olive leaves and branches, these are dried with hot air. This technique has a crucial role in the process because if the operating conditions are not optimal or appropriate, a huge loss of the total phenol content occurs. To model the process and minimize the losses of total phenols, the optimal operating conditions determined in Erbay et al.^51^ are employed. In this way, to obtain the moisture content below 6%, the dryer must operate at 53.43 °C.^51^ In the model, it is assumed that the olive leaf temperature is constant across the dryer and the air temperature can decrease by 3 °C. Thus, it is possible to compute the airflow necessary to dry the olive leaves. The energy balance developed in the process of drying is presented below
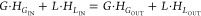
8where
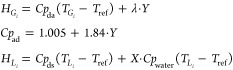
9To compute the heat capacity of the leaves, their composition and corresponding heat capacities are used^52^
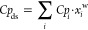
10
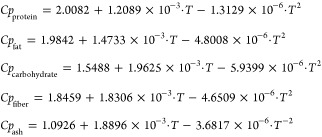
11According to the information collected, for this operating capacity, the investment cost of the dryer is around $15 000, while the operation and maintenance cost is a function of removed water in the leaves and takes a value around 9.82 × 10^–5^ M€/(kg H_2_O/h)^53^
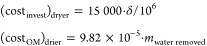
12After completing the drying process of the leaves, they are fed to the milling process and then the powder is stored. The cost of the crushing machine varies between $60 000 and $145 500. Due to the lack of information about this specific process, to be on the safer side, a higher cost is used. With regard to operation and maintenance cost, the cost of the energy consumed according to the specifications is assumed, taking a value of around 6.3 kW per ton of olive leaves milling per hour (t/h)^54^
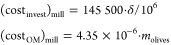
13

### Pressurized Liquid Extraction (PLE) for Phenol Recovery

Lama-Muñoz et al.^21^ developed correlations to compute the extraction yield of the soluble solid content from the liquid phase as well as the oleuropein content as a function of temperature, moisture, and ethanol concentration. The correlations shown in eq 14 are used to compute the extraction yield and the content of oleuropein in the olive leaves
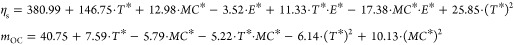
14The parameters T*, MC*, and E* take values between −1 and 1. To apply these values and boundaries in the model, a variable change is presented for the moisture content, temperature, and the ratio between ethanol and water. The boundaries applied in these correlations correspond to 60–80% of ethanol at 70–190 °C and 4.70–22.60% moisture contents, as given in eq 15
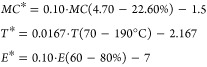
15In this technique, the ratio between the solvent and the olive leaves should be around 7 and is lower than the other techniques.^30^ The losses of solvent in the process are considered to be around 2% of the solvent fed.

Based on the work of Osorio-Tobón et al., a surrogate model is developed to calculate the investment cost of the pressurized liquid extractor as given by eq 16 ^55^
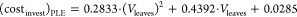
16To obtain a maximum range of extraction, the time and number of cycles in the process are 5 min and 1 cycle, respectively.^21^ To obtain a continuous process, two extractors are used. While one extractor is operating, the other one is cleaned and refilled with powdered leaves. Taking into account these considerations, the extractor capacity is computed through eq 17
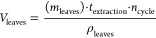
17The density of the powdered olive leaves is considered to be around 1510 kg/m^3^.^56^

The operation and maintenance cost are computed as a function of ethanol losses in the process, considering the cost of the ethanol to be around 0.42 €/kg.^57^

### Purification of Oleuropein Content

Two methods have been used to purify the oleuropein, membrane modules^11^ and adsorption resins,^58^ and only one is used.

### Membrane Modules

The technique of membrane modules employed in the model consists of two membranes (to the right in the bottom of Figure 3): an ultrafiltration membrane and a nanofiltration membrane. The treatment of membranes to purify oleuropein is similar to that of alperujo.

The first step is to increase the pressure with a pump until the transmembrane pressure (Δ*P*) generated reaches 1 bar. Having reached that pressure, the solution is sent to the ultrafiltration membrane, whose characteristics are shown in Table 5. The permeate obtained from the ultrafiltration membrane is sent through another pump to the nanofiltration membrane. In this pump, the transmembrane pressure must reach 9 bar. The product obtained consists of other phenols, flavonoids, and carbohydrates. The process to compute the investment cost and the operation and maintenance cost are the same as in the previous case.

**Table 5:** Properties of Membranes^11^

	*J*_p_ (L/(m^2^·h))	η_oleuropein_ (%)	η_soluble_ (%)
ultrafiltration membrane	27.5	32.81	37
nanofiltration membrane	50	100	95

### Selective Adsorption of Oleuropein

The extract obtained in the pressurized liquid extraction process is filtered through a filter under pressure to recover ethanol (see the units to the bottom left corner of Figure 3). Thus, ethanol is collected and recycled to the previous step. The ethanol losses are considered to be around 1%. The extract without ethanol is diluted with water, generating a ratio of (grams of extract per liter of water) around 100. After the adsorption process is completed, the resins are washed with distilled water and subjected to the desorption process. To carry out the desorption process, 40% ethanol in water mix^58^ is added to the resins, promoting the desorption of oleuropein. To remove the water, the obtained extract is percolated through a 0.45 μm filter.^15,58^ The resin employed is Amberlite XAD-7HP, recommended by Şahin et al. for the recovery of oleuropein from olive leaves.^58^Table 6 shows the main characteristics of the resin employed in the process.

**Table 6:** Properties of Amberlite XAD-7HP Resin

		References
*Q*_e_ (mg/g resin)	97.9	(58)
ρ (g/mL)	1.05	(59)
price (€/kg)	5.00	(59)

The yield of oleuropein recovery in the process of adsorption is 91%, while the process of desorption is 97%. The adsorption process runs for 180 min, whose adsorption capacity corresponds to 97.90 mg of oleuropein per g of resin^58^ and 17.50 mg per g of resin for the rest of phenols. The adsorption capacity is used to determine the amount of resin that is necessary for the operation of the process (eq 18)
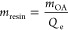
18To compute the cost of the process, the filter under pressure and the pressurized liquid extractor are considered. The filter under pressure is assumed to be a vessel, and the cost is estimated as follows^60^
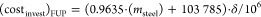
19The weight of the vessel is determined using eq 20 ^61^
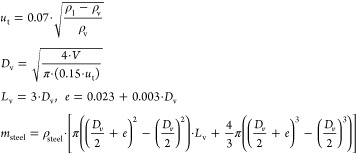
20The cost of the adsorption–desorption process is estimated using the price of the XAD resin and vertical vessels. The mass of resin employed in the process is determined as a function of the adsorption capacity of the adsorbent and the amount of sorbent. The size of the vessel should be such that it has to be able to contain the bed of resins. An overdesign factor of 10% is used for safety reasons and in the case of bed expansion (eq 21)^62^
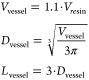
21The cost of resin XAD is considered to be 2000 €/t,^59^ and to estimate the cost of the vessel, a correlation obtained from Matche (eq 22) is used^60^
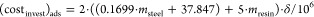
22The change of resin along the campaign is considered as operation and maintenance costs. One change per campaign is assumed to be on the safer side.

### Energy Recovery

Two of the products obtained in the recovery of phenols are solid compounds made of cellulose or lignin. To improve the sustainability of the process, where the pretreatments are energy-intense, the solids obtained are employed to produce energy and generate the steam used in the pretreatment step.

The first aim is to generate the steam needed in the pretreatment stage and heat the air in the dryer; the rest of the wastes is used in the production of electricity. To compute the energy required to generate the steam, the type of pressure is defined. A medium-pressure steam (2.7 MPa and 503.15 K)^63^ is produced. Therefore, based on these parameters and the amount of steam needed, which was fixed by the characteristics of the pretreatment, the energy required is calculated using eq 23
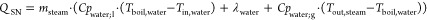
23Taking into account that the energy efficiency toward power in a Rankine cycle is around 40% (η_SN_), the energy needed in this process is computed as eq 24
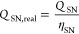
24As previously mentioned, the sources employed to generate thermal and electrical energies are bones and orujillo whose calorific values are 4400 and 4100 kcal/kg, respectively.^64^ The efficiency of the boiler to produce thermal energy is assumed to be 80%. The analysis of the energy produced from these wastes is carried out separately since alperujo is used for 4 months and olive leaves are used for the rest of the year. To compute the power generated, two boilers and two turbines are modeled with the characteristics of the wastes.

The process considered to produce electricity is a regenerative Rankine cycle with reheating. This cycle is modeled unit by unit like in the previous work, considering mass and energy balance and the detailed thermodynamics.^44^

The cost of this section of the facility is computed through the following surrogate models^65^
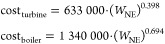
25

### Solution Procedure

#### Selection of Techniques: Superstructure Optimization

The aim of the process is the recovery of the added-value products together with the use of residues to produce power from the wastes of the olive oil production. Thus, the objective function is a simplified annual profit, considering a life cycle of 20 years, as presented in eq 26, where the annualized cost of the units involved allowed for the fixed costs of selecting a unit to be considered, while the operating costs consider the use of utilities involved in the processing of the waste
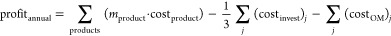
26Within the superstructure, the purity of the products depends on the selected technique. As a result, different prices are assigned to the products of each of the alternative path, as shown in Table 7.

**Table 7:** Price of Different Phenols Obtained

	obtained from	price (€/kg)	reference
hydroxytyrosol	adsorption	300	(66)
tyrosol	adsorption	100	(67)
hydroxytyrosol/tyrosol	membrane	85	(68)
oleuropein	adsorption	850	(69)
oleuropein	membrane	45	(70)

Two operating periods are considered: the recovery phenols from alperujo operate for 4 months, while the recovery phenols from olive leaves and branches work along the rest of the year. The optimization is subjected to the models of all of the unis described in the Superstructure Model Formulation section representing an NLP problem that consists of around 1818 equations and 2508 variables. The model is formulated in GAMS and solved using a multistart optimization approach with CONOPT as the preferred solver. No global optimum is claimed.

### Cost Estimation

After the optimization of the superstructure, the operation and maintenance costs are computed based on Sinnot and Towler’s factorial method.^71^ The investment cost of the facility is based on the unit’s costs, estimated using the equations presented along the text. The total investment is estimated using Lang factors for a facility that processes fluids and solids.

With regard to the production costs, the variable cost includes the cost of raw materials and utilities, while the fixed cost includes the cost of maintenance, operating labor, laboratory cost, supervision, plant overheads, capital charges, insurance, local taxes, and royalties. The methodology employed to develop this cost is described by Sinnott and Towler.^71^

## Results and Discussion

As a case study for the analysis, 99 227.4 tons of olives gathered per campaign are considered. In the oil production, 800 kg of alperujo is generated per ton of olives, and leaves and branches generated represent around 5% of the mass of the total amount harvested. Results obtained after the selection of products and the optimization process are shown in the next subsections.

### Portfolio of Products

The first part of the results consists of the application of the indicators developed to select the product(s) to be obtained. In the case of study, only two indicators can be applied, on the one hand, the economic potential indicator and, on the other hand, the energy indicator regarding the rupture of the raw material in smaller parts. For the indicator concerning solvents, in the majority of the processes, ethanol is employed, which is considered as a green solvent.^72^ For developing the methodology, a particular composition of olive pomace and leaves and branches is taken, allowing the characterization and selection of these wastes.

### Alperujo

The composition of phenols from alperujo is shown in kilograms per cubic meter (Table 8). To compute the fraction of each one in the alperujo, the density of the olive pomace density is assumed to be 1.63 g/cm^3^.^73^ To estimate the energy indicator, it is necessary to determine the original source of the phenols. Two alternative sources are identified. We consider the natural source, the phenols already available within the waste. Alternatively, they can be produced from the breaking down of longer molecules. In this part of the study, only two phenols can be obtained from the different sources: hydroxytyrosol and tyrosol. Oleuropein, verbascoside, and hydroxytyrosol 4-β-d-glucoside can be broken down to form hydroxytyrosol. In the case of tyrosol, it can be obtained from oleuropein and natural sources. Without experimental data, it is not possible to determine the proportion of rupture of each molecule. To compute the energy indicator, we assume that each source can generate the same amount. In other words, to calculate the indicator for the hydroxytyrosol, we consider 25% of each source, and in the case of tyrosol, we consider 50%. To consider the same source for the price of the products in all cases, the price at lab-scale from Sigma-Aldrich’s web page is used (see Table 8).

**Table 8:** Results of Indicators Applied in the Main Phenols of Alperujo

	amount of phenols (kg/m^3^)	amounts of phenols in alperujo (%)	cost (€/mg)	molecular weight (g/mol)	η_profit_	η_energy_	standardization of η_profit_
3,4-dihydroxyphenylglycol	0.157	0.00963	10.26^74^	170.16	1.39 × 10^3^	1	0.12
hydroxytyrosol	0.540	0.03313	25.41^75^	154.16	1.18 × 10^4^	2.652	1.00
tyrosol	0.122	0.00748	13.1^76^	138.16	1.62 × 10^3^	2.456	0.14
vanillin	0.076	0.00466	2.45^77^	152.15	1.60 × 10^2^	1	0.01
*p*-coumaric acid	0.032	0.00196	6.90^78^	164.16	1.90 × 10^2^	1	0.02

Table 8 shows the results of the indicators that can be applied in this case of the study. To rank the products, the economic potential indicator shows that the best phenols to recover are hydroxytyrosol, tyrosol, 3,4-dihydroxyphenylglycol, *p*-coumaric acid, and vanillin. In this way, the best option for recovery is hydroxytyrosol.

Regarding the energy indicator, since there is a possibility to obtain hydroxytyrosol and the tyrosol from other phenols, their indicator shows higher values with respect to other phenols.

In the view of the above results, hydroxytyrosol is the best option for recuperation but the literature suggests considering both hydroxytyrosol and tyrosol.

### Leaves and Branches

In the case of olive leaves and branches, the calculation procedure is the same as in the previous case (see Table 9 for the properties of the raw material). The main difference is that the composition of olive leaves is given on a dry basis. The amount of water is around 49.7% based on the literature and that is the value assumed for the residue.^16^

**Table 9:** Results of Indicators Applied in the Main Phenols of Olive Leaves and Branches

	amounts % (dry basis)	amounts %	cost ($/mg)	*M*_w_ (g/mol)	η_profit_	η_energy_	standardization of η_profit_
oleuropein	11.5	5.78	18.5	540.51^79^	1.36 × 10^8^	1	1.00
luteolin-7-glucoside	0.319	0.16	40.47	448.38^80^	8.28 × 10^6^	1	0.06
apigenin-7-glucoside	0.134	0.07	31.4	432.38^81^	2.70 × 10^6^	1	0.02
verbascoside	0.086	0.04	31.45	624.59^82^	1.73 × 10^6^	1	0.01
quercetin	0.026	0.01	0.0051	302.24^83^	8.49	1	0.00

In this case, it does not make sense to compute the energy indicator because all phenols are found in the waste. As a result, the compound selected for the extraction in olive leaves is oleuropein. In this case, only one compound is selected because it is the major compound in leaves and generates the larger economic potential indicator by comparison.

### Process Design: Selection of Techniques to Recover Phenols

**Figure 4:**
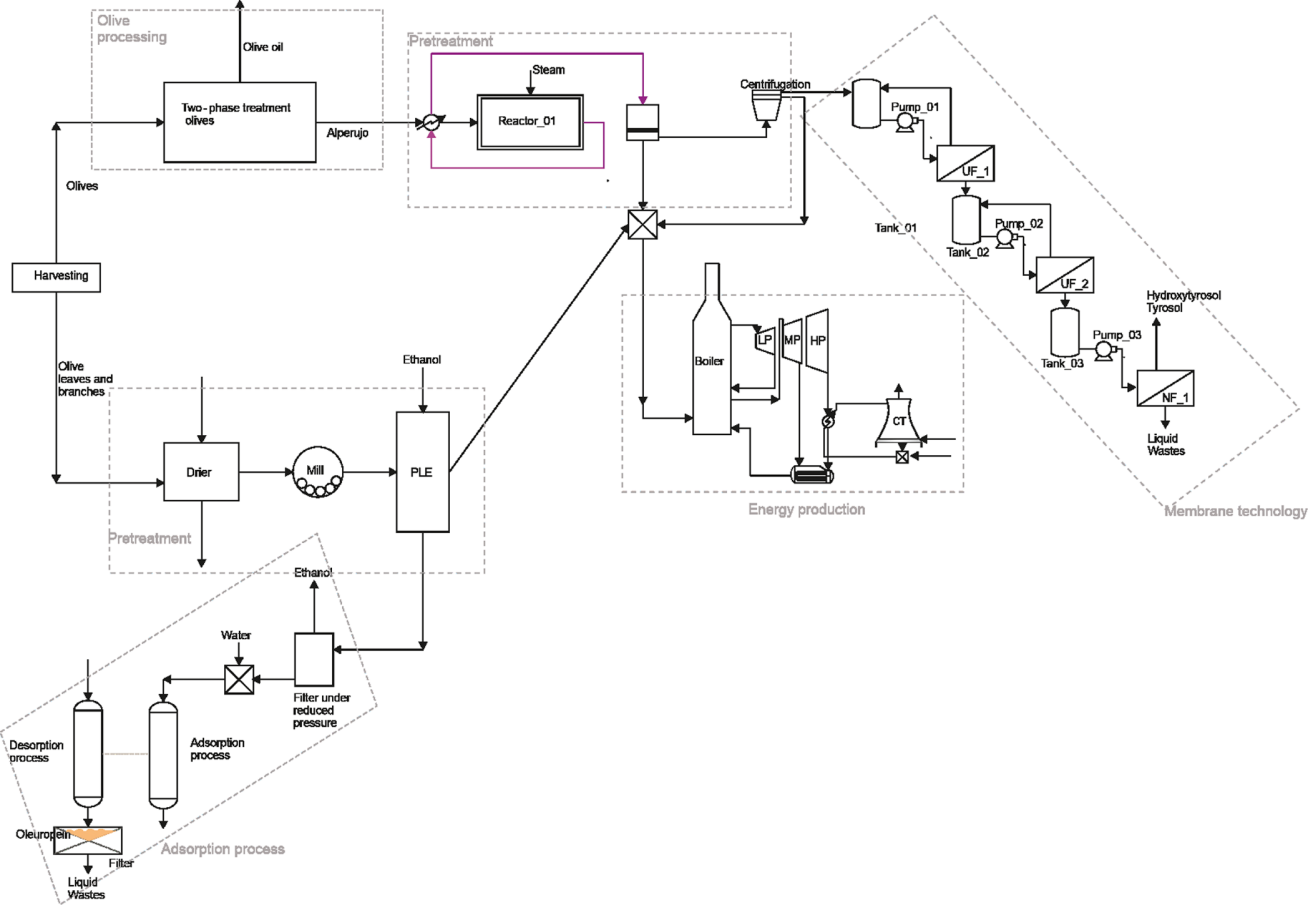
Optimal flowsheet for the processing of olive’s waste. Solution of the superstructure optimization.

The result of the optimization of the superstructure is presented in Figure 4. The use of membrane module is selected to recover phenols from alperujo, while for processing olive leaves and branches, the adsorption technique is one of the choices.

From the economic point of view, the recovery of phenols from alperujo should be done using the membrane module. The main reason behind this is the high cost of the resin employed to separate and recover hydroxytyrosol and tyrosol separately. It should be noted that in this case of using the membrane separation, the product generated is a mix of phenols composed mainly of hydroxytyrosol and tyrosol, but it is cheaper compared to the use of adsorption and the production of both separately does not compensate for the additional cost. The mix of phenols obtained through this process is around 62% of hydroxytyrosol, 26.7% of tyrosol, and the rest of soluble compounds like other phenols or carbohydrate-soluble. Nowadays, this type of mix of phenols has wide use in different sectors such as pharmaceuticals, food products, and cosmetics, among others.

**Figure 5:**
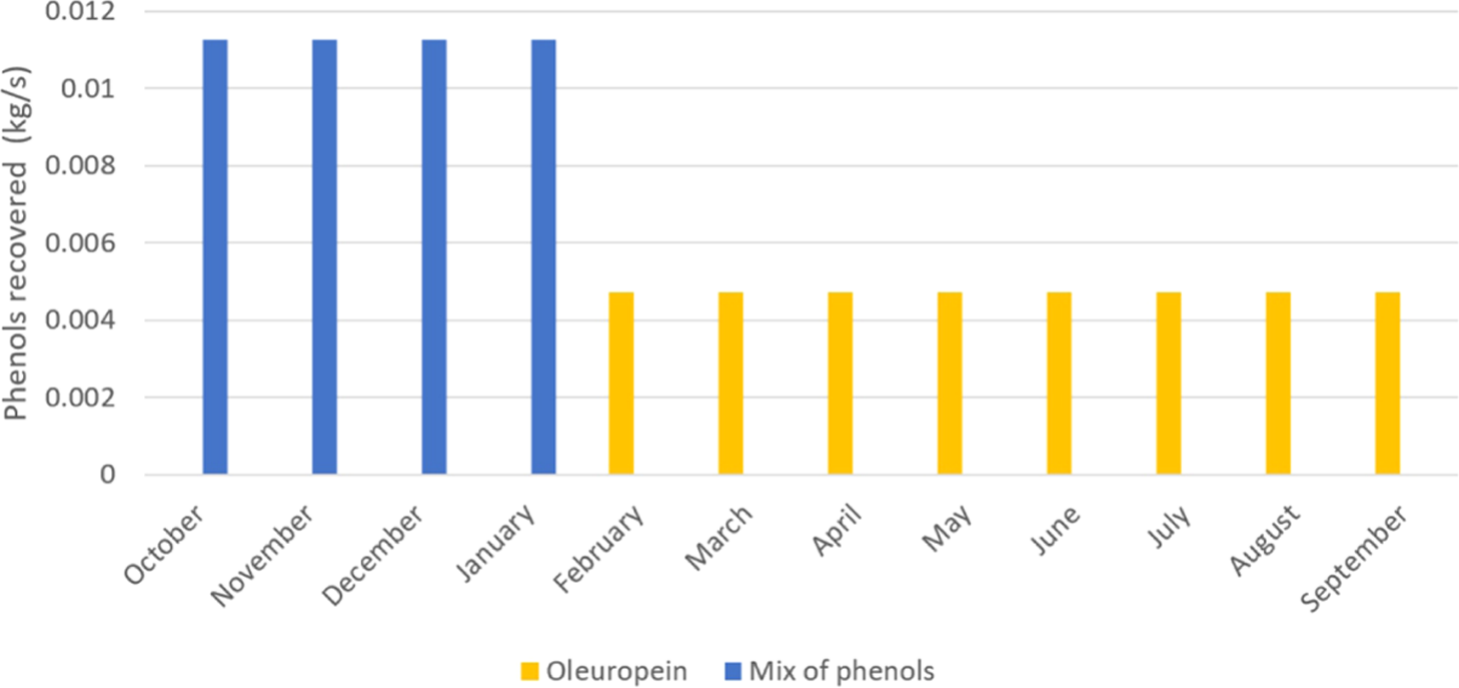
Summary of the products obtained over a year of operation from alperujo and the leaves and branches.

In the case of olive leaves and branches, it is more profitable to use adsorption for the recovery of phenols. Since the phenol recovered is different from that of alperujo, the type of resin employed in the process must also be different, resulting in a cheaper cost. The adsorption technique allows the production of oleuropein with 85.5% purity. The quantity of phenols per month is shown in Figure 5. Note that the production of olives lasts 4 months, which results in the production of alperujo from processing the waste, while the rest of the year, the leaves and branches are processed to obtain the oleuropein.

### Economic Evaluation

Using the correlations developed for each one of the units as a function of their size, the cost of each unit is computed. The investment in units adds up to 18.22 M€. Table 10 shows the detail of the processing lines for alperujo and the leaves and branches. The total investment cost of the plant is around 110.23 M€.

**Table 10:** Investment Cost of Units of Alperujo and Olive Leaves and Branches

	treatments	investment cost (M€)
alperujo	pretreatment	0.524
	membrane technology	0.597
	UF 1	0.306
	UF 2	0.276
	NF 1	0.014
	power technology	9.919
	turbine	1.75
	boiler	7.93
leaves and branches	dryer	0.01275
	mill	0.1455
	filter under pressure	0.089
	PLE	0.039
	resin adsorption	6.319

As shown in Table 10, the share of the alperujo pretreatment used for the separation of the liquid and solid phases represents 46.74%, while the rest of the investment cost corresponds to the membrane module technology. In the case of olive leaves and branches, only around 3% correspond to the investment in the pretreatment. The investment costs for the turbine and the boiler are around 1.75 and 7.93 M€, respectively. Note that the power plant represents more than half the investment in units. Note that because the larger solid production comes from the alperujo, there is a need to design the power island based on that production capacity.

**Figure 6:**
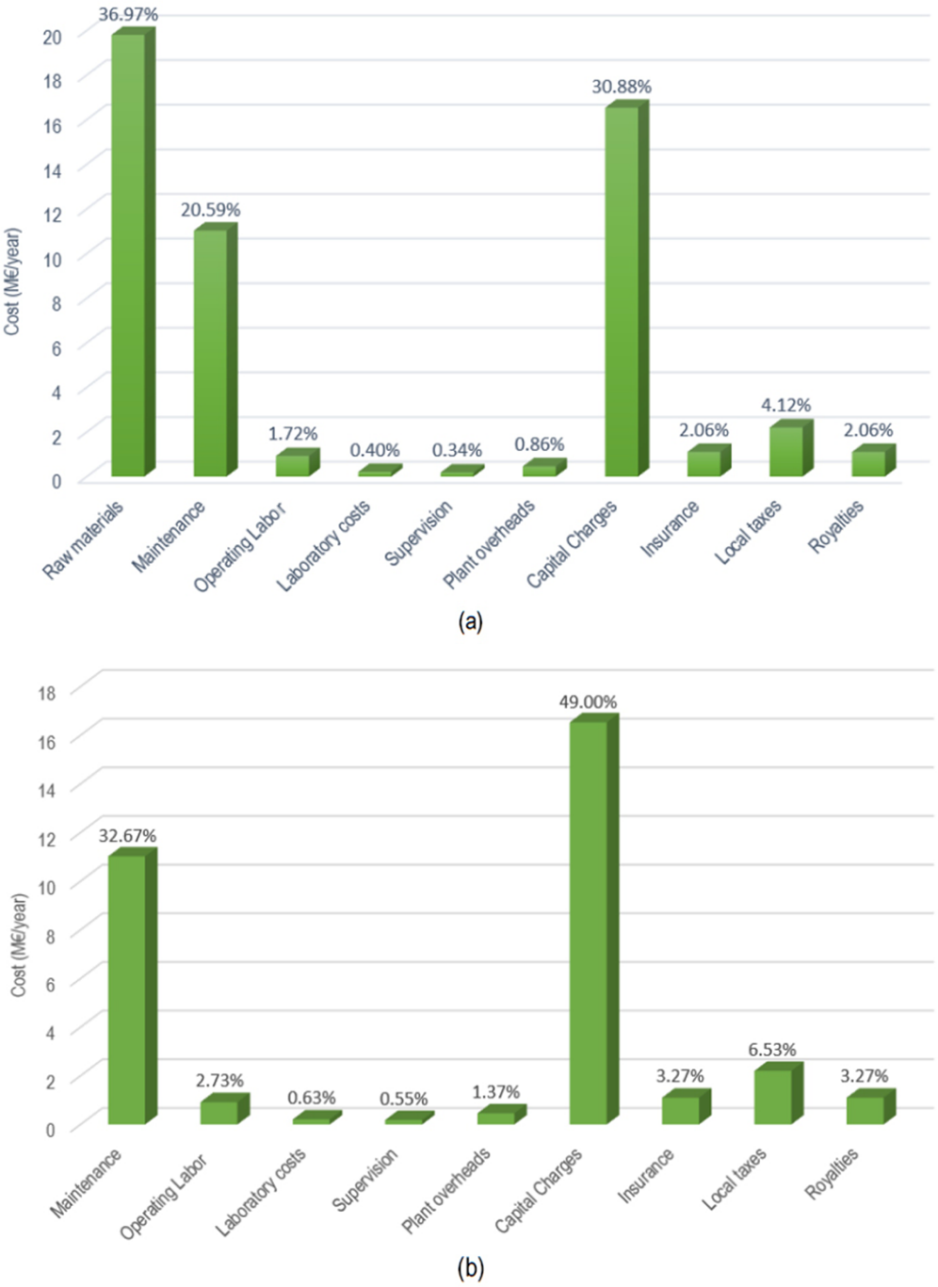
Total operation and maintenance costs: (a) considering raw materials and (b) not considering raw materials.

The operation and maintenance costs are described in the Solution Procedure section. Typically, this methodology considers the cost of utilities like electricity and steam as variable costs. However, in this case, it has not been considered due to the fact that the facility has the capacity to generate them. Two alternative solutions are presented, whether the raw materials are received at a zero cost or if they are purchased. In the latter case, prices of €249 per ton of alperujo^84^ and €6 per ton of olives and branches are considered. Figure 6a shows the first alternative, and Figure 6b shows the second one. The operation and maintenance costs without raw materials adds up to 33.75 M€/yr, while in the case where raw materials are considered, the costs reach a value of around 53.54 M€/yr. The cost increases by 61% in the case the raw materials do not belong to the facility. As shown in Figure 6a, the cost of raw materials reaches 37% of the operation and maintenance costs followed by capital changes and maintenance costs. In Figure 6b, costs have the same order, the capital changes being more expensive followed by maintenance and local tax costs.

Benefits from phenols correspond to around 104.7 M€/yr. Using eq 26, we compute profit, considering benefits from phenols, annualized investment cost, and the operation and maintenance cost defined in Figure 6. In the case of purchasing the raw materials, the profit reaches 14.5 M€/yr, while in the other case, the profit achieved is around 34.3 M€/yr. The return on investment in the case of raw materials accounted is around 16.02%, while in the case of raw materials not accounted, it is approximately 48.61%.

### Energy Evaluation

**Figure 7:**
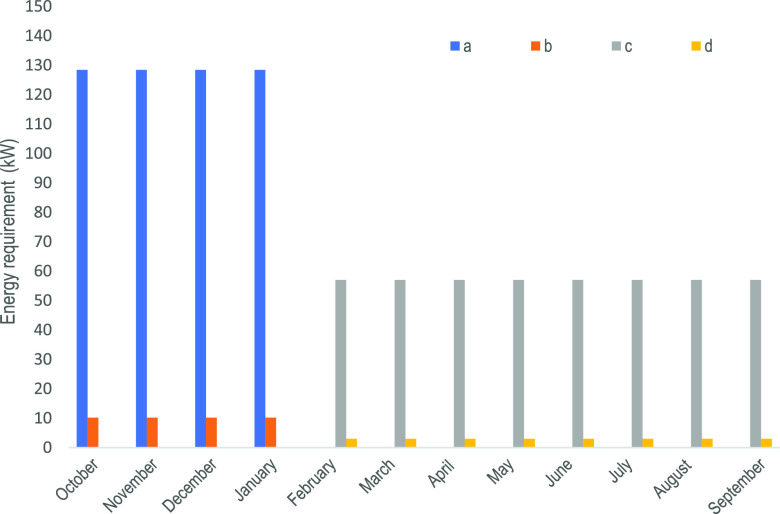
Monthly energy requirements. (a) Thermal energy required by alperujo, (b) electric energy required by alperujo, (c) thermal energy required by olive leaves and branches, and (d) electric energy required by olive leaves and branches.

Solid wastes are used to generate electric energy and thermal energy. Thermal and electrical energy requirements annually by the part of the facility to recover phenols from alperujo is around 140 kW on a monthly basis, of which 92.6% corresponding to thermal energy. While in the part of recovery, oleuropein from olive leaves and branches achieves 60 kW monthly, 95% corresponding to thermal energy. To better understand the operation of the facility, the monthly thermal and electrical energy requirements of alperujo and olive leaves and branches are shown in Figure 7.

Once the thermal energy requirement is fulfilled, the rest of the solid wastes are transformed into electric energy by the power plant. Results show that from the alperujo residues, 12.95 MW can be produced, while from the leaves and branches, the power can only be 312 kW. In view of these results, the power plant may only operate for 4 months because, the rest of the months, the power island of the facility should operate at such a low load that it will cause a problem for the units.

**Figure 8:**
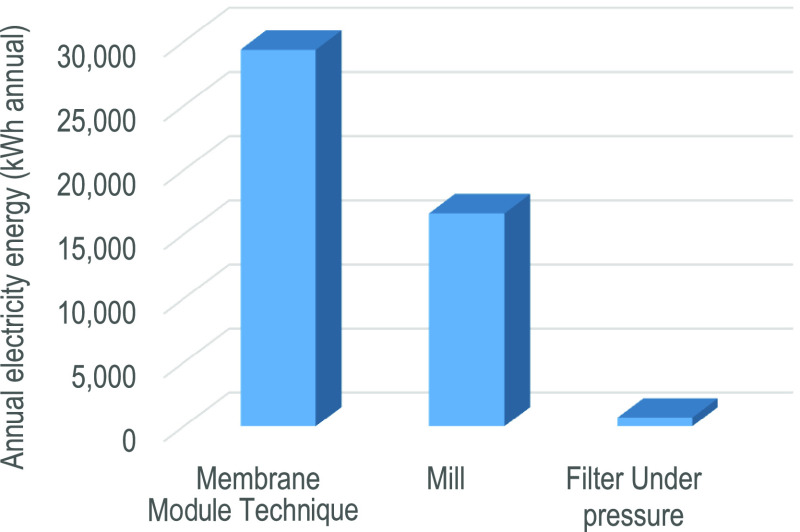
Annual electric energy consumption by techniques.

The electric energy consumed by the units corresponds to 10.2 kW during the alperujo treatment (4 months) and 3 kW during the leaf and branch processing (8 months), a total of 46,711 kWh (see Figure 8). Since the power plant capacity installed in the facility is around 12.95 MW and 312 kW for each of the operating periods, it is not necessary to buy this type of utility. In this way, the facility, or the part of recovery phenols of the facility, is capable of supplying on its own.

In case the recovery of phenols becomes part of the olive oil generation facility, the energy consumption of the main section producing the olive oil should also be analyzed. Considering the production capacity of this facility and data from the literature, the annual energy consumed by the olive oil production is around 3920 MWh.^85^ Therefore, the power plant using the residue from the alperujo, with a total energy production of 37 296 MWh, would be able to supply energy at this part of the facility.

## Conclusions

In this work, a systematic methodology has been developed for the screening and designing of multiproduct facilities associated with the WEF nexus. It combines hybrid heuristic-based, metric-based, and superstructure optimization approaches to identify the promising high added-value products so as to put together a superstructure for the design of an integrated facility that makes the most of waste and valuable products toward a sustainable biorefinery. Novel indicators have been developed to identify value-added products. The methodology has been applied to the waste from olive trees, olive leaves and branches, and alperujo from olive oil production. Value-added products of these wastes include phenols that can be used in the pharmacy, cosmetic, or food industries.

The results show that the optimal portfolio of products corresponds to the recovery of phenols, hydroxytyrosol, and tyrosol from alperujo and oleuropein from olive leaves and branches based on the economic indicator, which is the largest. After the selection of the products to be obtained, a superstructure is formulated and optimized. As a result of the optimization, pretreatment and membrane module technique should be used from alperujo to recover a mix of phenols, while in the case of olive leaves and branches, a dryer followed by a mill, a pressurized liquid extractor, a filter under pressure, and resin adsorption are used. The final economic evaluation is carried out, wherein the profit comes to 14.5 M€ annually in case this process is integrated into an olive oil production facility and 34.3 M€ annually if the raw materials have to be purchased. Regarding energy, solid waste is used to generate the utilities employed in the recovery process. A power plant is integrated into the facility where the power is around 12.95 MW, which is sufficient for the recovery process.

## Nomenclature

cost_boiler_: cost of boiler (€)
(cost_invest_)_ads_: inversion cost of adsorption (M€)
(cost_invest_)_dryer_: inversion cost of dryer process (M€)
(cost_invest_)_FUP_: inversion cost of filter under pressure (M€)
(cost_invest_)_mill_: inversion cost of mill (M€)
(cost_invest_)_PLE_: inversion cost of pressurized liquid extractor (M€)
(cost_invest_)_PT_: inversion cost of pretreatment (M€)
(cost_invest_)_RA_: inversion cost of resin adsorption (M€)
(cost_OM_)_dryer_: operation and maintenance cost of dryer (€)
(cost_OM_)_Mill_: operation and maintenance cost of mill (M€)
(cost_OM_)_PT_: operation and maintenance cost of pretreatment (M€)
(cost_OM_)_RA_: operation and maintenance cost of resin adsorption (M€)
cost_turbine_: cost of turbine (€)
*Cp*_ash_: heat capacity of ash (kJ/(kg·K))
*Cp*_carbohydrates_: heat capacity of carbohydrates (kJ/(kg·K))
*Cp*_da_: heat capacity of dry air (kJ/(kg·K))
*Cp*_ds_: heat capacity of dry leaves (kJ/(kg·K))
*Cp*_fat_: heat capacity of fat (kJ/(kg·K))
*Cp*_fiber_: heat capacity of fiber (kJ/(kg·K))
*Cp*_protein_: heat capacity of protein (kJ/(kg·K))
*D*_v_: diameter (m)
*D*_vessel_: diameter (m)
*e*: thickness (m)
*E*: ethanol concentration
*E**: ethanol concentration (−1; 1)
*G*: dry airflow (kg dry air/s)
*H*_Gi_: enthalpy of air under “*i*” conditions (kJ/kg)
*H*_Li_: enthalpy of solid under “*i*” conditions (kJ/kg)
*L*: dry solid flow (kg dry leaves/s)
*L*_v_: tank length (m)
*L*_vessel_: tank length (m)
MC: moisture content (%)
*MC**: moisture content (−1:1)
*m*_leaves_: mass flow of leaves (kg/s)
*m*_resin_: mass of resin (kg)
*m*_steel_: mass of steel (kg)
*m*_steam_: mass flow of steam (kg/s)
*m*_treat_: treated mass (kg/yr)
*m*_t_: treated mass (m^3^/yr)
*m*_OC_: oleuropein content (kg oleuropein/kg dry leaves)
*m*_OA_: mass of oleuropein adsorbed (kg soluble/kg dry leaves)
*m*_olives_: mass flow of olives (kg/h)
*m*_water removed_: mass flow of water removed (kg/h)
*n*_cycle_: number of cycles
*T*: temperature (°C)
*T**: temperature (°C)
*t*_extraction_: extraction time (s)
*u*_t_: terminal velocity (m/s)
*V*: steam volume (m^3^)
*V*_leaves_: extraction vessel capacity (m^3^)
*V*_resin_: volume of resin (m^3^)
*V*_vessel_: volume of vessel (m^3^)
*W*_NE_: net electric energy power output (MW)
*X*: humidity of leaves (kg water/kg dry leaves)
*x*_i_^w^: mass fraction of *i* component
*Y*: specific moisture (kg water/kg dry air)
δ: conversion euro dollar (0.85 $/€)
ρ_leaves_: leaf density (kg/m^3^)
ρ_l_: liquid density (kg/m^3^)
ρ_v_: vapor density (kg/m^3^)
ρ_steel_: steel density (kg/m^3^)
